# Development of Laser Scanner for Full Cross-Sectional Deformation Monitoring of Underground Gateroads

**DOI:** 10.3390/s17061311

**Published:** 2017-06-07

**Authors:** Qianlong Yang, Zhenyu Zhang, Xiaoqian Liu, Shuqi Ma

**Affiliations:** 1 State Key Laboratory of Coal Mine Disaster Dynamics and Control, Chongqing University, Chongqing 400044, China; cqyql@cqu.edu.cn (Q.Y.); liuxq@cqu.edu.cn (X.L.); 2 Laboratory and Equipment Managing Division, Chongqing University, Chongqing 400044, China; 3 School of Civil and Environmental Engineering, Nanyang Technological University, Singapore 639789, Singapore; sqma@ntu.edu.sg; 4 Key Laboratory of Transportation Tunnel Engineering, Ministry of Education, School of Civil Engineering, Southwest Jiaotong University, Chengdu 610031, Sichuan, China

**Keywords:** laser sensor, deformation monitoring, underground gateroad

## Abstract

The deformation of underground gateroads tends to be asymmetric and complex. Traditional instrumentation fails to accurately and conveniently monitor the full cross-sectional deformation of underground gateroads. Here, a full cross-sectional laser scanner was developed, together with a visualization software package. The developed system used a polar coordinate measuring method and the full cross-sectional measurement was shown by 360° rotation of a laser sensor driven by an electrical motor. Later on, the potential impact of gateroad wall flatness, roughness, and geometrical profile, as well as coal dust environment on the performance of the developed laser scanner will be evaluated. The study shows that high-level flatness is favorable in the application of the developed full cross-sectional deformation monitoring system. For a smooth surface of gateroad, the sensor cannot receive reflected light when the incidence angle of laser beam is large, causing data loss. Conversely, the roughness surface shows its nature as the diffuse reflection light can be received by the sensor. With regards to coal dust in the measurement environment, fine particles of floating coal dust in the air can lead to the loss of measurement data to some extent, due to scattering of the laser beam.

## 1. Introduction

With continuous extraction of coal resources, mining has gradually reached deeper geological strata [1,2,3]. Under such deep geological environments, underground gateroads tend to frequently produce large and asymmetric deformation [4,5]. In conventional deformation monitoring of tunnel and underground excavation, the convergence between roof and floor and the convergence between two side walls are measured, while the deformation monitoring of the rest of excavation profile is seldom covered [6,7]. Furthermore, the geometric profiles of gateroads are variable in underground mining and rectangular, trapezoidal, and semi-circular arch gateroads are frequently used for different functional purposes and mine-site specific geological conditions [8,9,10,11]. Therefore, it is far from comprehensive to evaluate gateroad deformation based solely on deformation data monitored from limited points. This has stimulated the development of a full cross-sectional deformation monitoring system to ensure the safety of mining activities.

Convergence extensometers, measuring rods, and flexible rulers are traditionally used to manually capture the deformation of gateroads. However, their measurement precision is significantly influenced by a variety of practical factors, such as monitoring system installation and personnel operation. In another aspect, the traditional methods are based on relative distance measurement between two specific points, and fail to measure the asymmetric deformation pattern of gateroads. Alternatively, laser scanning technology is a non-contact distance measurement technique and shows great potential in such deformation measurements by recording the time difference between the laser’s emission and reflection after meeting the target surface [12,13].

In this study, a full cross-sectional deformation monitoring instrument was developed by combining a laser distance measurement method and computer processing technology. Firstly, development and application of the laser scanning technology in change detection and deformation monitoring is briefly reviewed. Then the development of a full cross-sectional deformation monitoring system is introduced step-by-step. Later on, the potential impacts of wall flatness, wall roughness, geometric profiles of cross section, and the dust concentration on the performance of the developed laser scanner are evaluated.

## 2. The Terrestrial Laser Scanning in Change Detection and Deformation Measurement

### 2.1. Overiew of Terrestrial Laser Scanning

The non-contact Terrestrial Laser Scanning (TLS) can not only complete the measurements in a very short time, but also harvest a substantial amount of point data. Therefore, TLS is an efficient method in change detection and deformation measurement, especially for remote and large range measurement. Due to these advantages, recently it has gained increasing attention in a wide variety of engineering fields, such as surveying, structure monitoring, civil, and mining engineering [14,15,16]. So far, static and mobile laser scanning systems have been developed for change detection and deformation measurement, such as terrestrial laser scanning (TLS) [17,18,19]. Three measurement principles are mainly used in change detection and deformation measurement with TLS: triangulation, time-of-flight, and structured light and photogrammetry [20].

In contrast, conventional techniques at a limited number of points can identify the change or deformation up to millimeter level, while the measurement of TLS varies from ±2 mm to ±25 mm, subject to the impact of specific instrument, observation condition and data processing model [14]. Many studies have been conducted to improve the measurement accuracy and explore its potential use in wider fields. Regarding the pre-process of data with TLS, Höfle and Pfeifer [21] developed data- and model-driven methods to reduce the intensity data variation due to spherical loss, and topographic and atmospheric effects. Bae et al. [22] used covariance analysis for edge and boundary detection from point cloud data. Pathak and Singh [23] modelled the impact of the morphology of the scanned surface using a response surface method, and identified two critical parameters influencing the accuracy of the scan model. For post-process of data points, Chen et al. [24] extracted six deformation parameters, including deformation magnitude and direction, using the improved Iterative Closest Point algorithm. Using least squares surface matching algorithm proposed by Gruen and Akca [25], Monserrat and Crosetto [26] developed a new procedure for deformation measurement, including steps of acquisition of the TLS data, registration of the point clouds, and deformation parameter estimation. In addition, repeated measurements were also recommended to improve the quality of raw data points in practical change detection and deformation measurement with TLS [14,15,26].

### 2.2. Application of TLS in Deformation Monitoring

As this study focuses on tunnel deformation monitoring with TLS, a brief review of the applications to this field is reported in this subsection. In addition, we also provide a quick overview on the application of TLS to related fields of civil engineering and geosciences, such as mining and landslides.

The capability of collecting high-density data clouds of TLS makes it popular in monitoring the deformation of long tunnels. Yoon et al. [27] developed a trial model scanner using the principle of time-of-flight, and further developed an algorithm to extract features of the concrete tunnel liner up to the accuracy of 5 mm. Fekete et al. [28] implemented the TLS to characterize discontinuity information of tunnels. Pejić [29] introduced an arbitrary georeferencing approach to reduce the geometric distortion of point clouds. Argüelles-Fraga et al. [30] discussed the impact factors of the laser scan process, and recommended to set the TLS equipment at the center of the tunnel to increase the incidence angle. Many application reports of TLS in tunneling and caves can be found in the literature [31,32,33].

With regards to its application in mining, Ghabraie et al. [34] used the TLS to study the strata mechanics behavior during earth source extraction in laboratory, and developed a physical modelling protocol for equivalent materials methodology. In the field scale, Lian and Hu [35] used TLS to evaluate the stability of surface high-voltage towers at different mining periods. Scaioni et al. [36] outlined a method to detect the change due to the deformation of rock face.

The TLS also has a wide range of applications in mapping landslides [37]. Razak [38] evaluated the suitability of airborne laser scanning-derived terrain models in mapping landslides and identifying morphological features of landslides. Using a 2D statistical normalized cross-correlation function, Travelletti [39] reported an approach to extract the 3D deformation from scanning at different epochs. With TLS, Jebur [40] optimized the landslide conditioning factors in landslide susceptibility analysis. Franz et al. [41] used targets to reduce the negative effect of high vegetation on the scan in landslide mapping. For the purpose of improving the accuracy of mapping landslides with TLS, Barbarella et al. [42] evaluated the uncertainty of monitoring data.

Overall, the application of TLS in deformation monitoring is rapidly expanding, and these applications not only include the fields mentioned above, but also cover the deformation monitoring of slopes [43,44], roads [45], cliffs [46,47], dams [48,49], buildings [50,51], bridges [52], etc.

## 3. Monitoring System Development 

**Figure 1:**
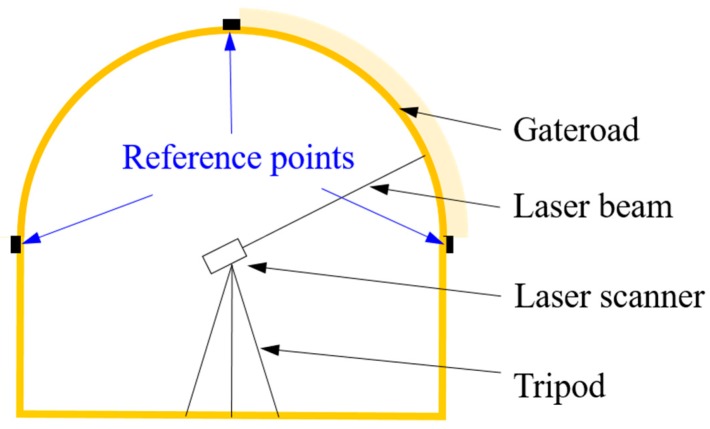
Deformation monitoring set-up in underground gateroads.

The developed full cross-sectional deformation monitoring system mainly consists of a laser scanner and visualization software package. Figure 1 shows the deformation monitoring set-up in underground gateroads. The reference points are designed to ensure that the generated Cartesian coordinate system in different measurements is exactly the same, in order to facilitate the deformation comparison at different time instances in engineering. The reference points are composed of three points falling in a spatial plane. With the laser distance measurement method, the full cross-sectional deformation of a gateroad is recorded and then transferred to the microcontroller. Finally, the full cross-sectional deformation of a gateroad between each two monitoring instants can be visually presented with the developed visualization software package.

**Figure 2:**
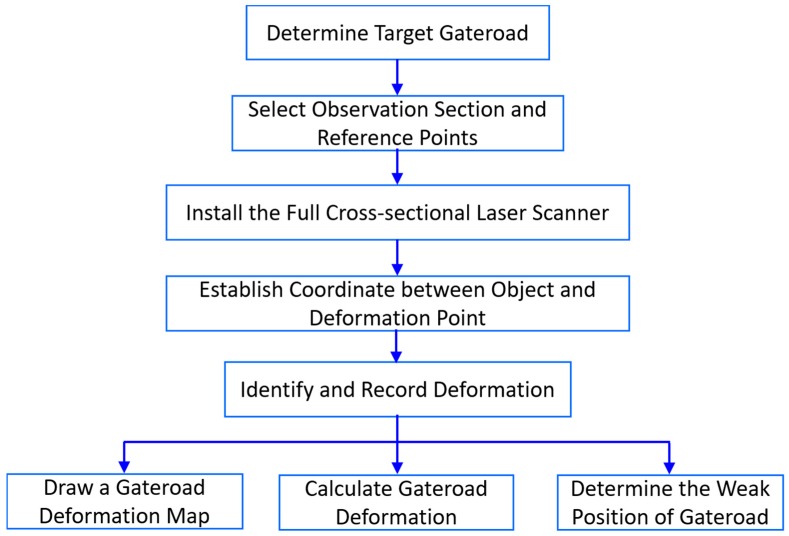
The flowchart of gateroad deformation monitoring with developed system.

Figure 2 shows a flowchart of deformation monitoring with the designed system. Prior to the first measurement of full cross-sectional geometrical profile of a gateroad, the observation locality needed to be determined, as well as three reference points. Subsequently, the laser scanner were set up in the field to measure the initial geometric profile of the gateroad. Then, repeated measurement were conducted according to practical requirements at different stages of mining. Finally, the measurement data was used to extract the deformation of the gateroad at different mining epochs, including plotting of a deformation map, extraction of gateroad deformation at a specific point, and determining the potential weak position of the gateroad.

### 3.1. Laser Scanner Design

The developed deformation monitoring system consists of a power module, central control processor, laser measurement module, data storage module, and data communication module of SD cards. The distance is measured with the multi-period pulse laser distance measurement method. The power module is in charge of power supply for the central control processor, laser measurement module, data storage, and communication module of SD cards. The central control processor controls the laser measurement module, data storage module, and data communication module of SD cards in the measurement process. The laser measurement module consists of a laser displacement sensor and driving device. The laser displacement sensor is composed of a beam projector and optical receiver. After scanning, the data was transferred to the data storage module with labeling. Finally, data was stored in an SD card via data communication module of SD cards.

**Figure 3:**
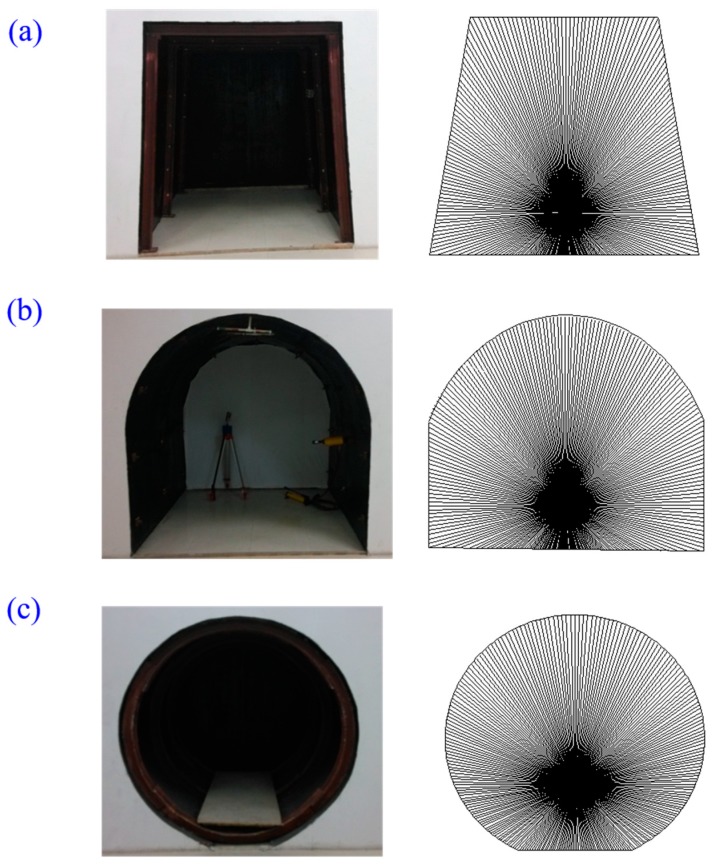
Schematic diagram and photos of gateroad deformation monitoring with laser scanner rotation: (**a**) trapezoidal gateroad; (**b**) semi-circular arch gateroad; (**c**) circular gateroad.

The key design aspect of the full cross-sectional deformation monitoring system is in controlling the laser scanner to realize rotation measurement. This was realized by 360° intermittent rotation of laser scanner driven by a motor controlled by an integrated chip. At each rotation step, the laser scanner rotates 1°27′45″. The laser scanner obtains the data from 256 points after 360° rotation, as shown in Figure 3. It takes approximately 2 s for the laser scanner to complete the distance measurement at each point.

### 3.2. Laser Displacement Sensor

The laser distance measurement sensor is composed of a laser emission module, laser receiver, clock module and data processing module. Functionally, it measures the time of a laser beam traveling from emission to the moment is it reflected back, and then processes the data to obtain the distance information with a clock module. Table 1 lists the parameters of the laser distance measurement sensor.

### 3.3. Visualization Software Package

The software package was developed to visually analyze the gateroad deformation at different time instants of engineering with Microsoft Visual Basic. In the software package, user proxy was designed to detect the legitimacy of software users, in order to avoid illegal users in the database. More importantly, the developed software package permits users to choose data of any specific gateroad section, and also supplies the missing point information by analyzing data error. With the selected data, the deformation of a specific section of gateroad at two different time instants can be visually plotted. Furthermore, the developed software package permits the exportation of the database for further engineering analysis.

## 4. Influence of Gateroad Characteristics on Measurement Data Loss

**Figure 4:**
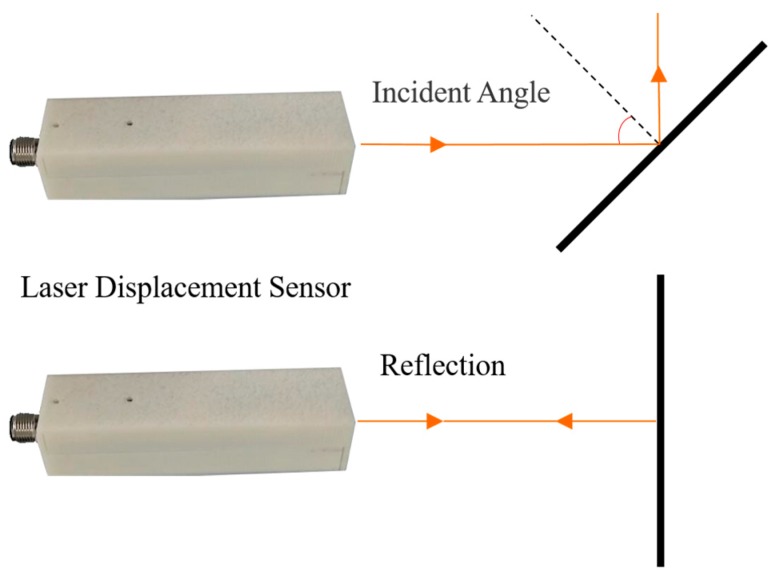
The schematic diagram of reflector dips effect.

In practical deformation monitoring, the measurement environment of underground gateroads tends to be complex due to site-specific geologic conditions, geometric profile, roughness of gateroad wall, and so on. Further, the rotation of laser distance measurement sensor changes reflection angle, as shown in Figure 4. As a consequence, the laser distance measurement sensor may fail to receive reflected light in the form of data loss. Here, the influence of complex environment of underground gateroads on the performance of developed full cross-sectional laser scanner was evaluated, including wall flatness, wall surface roughness, and geometric profile of gateroads.

### 4.1. The Influence of Surface Flatness on Data Loss

**Figure 5:**
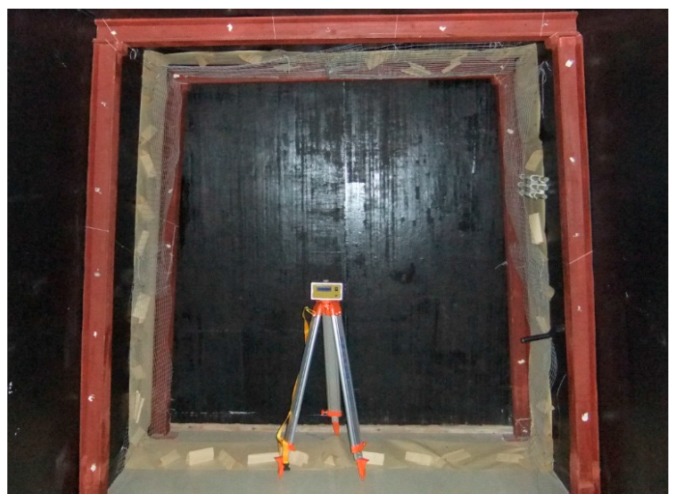
The surface flatness experiment of gateroads with developed deformation monitoring system.

Surface flatness denotes the degree of flatness of a surface. In underground gateroads, anchors of metal mesh, plastic nets, etc. are widely used for ground control, making gateroads surface to tend to have different degrees of flatness [2,53]. This can easily make the laser displacement sensor lose data. A flat trapezoidal surface, uneven trapezoidal surface, flat trapezoidal surface with metal mesh, and uneven trapezoidal surface with metal mesh were prepared to test their influence on the data loss in deformation monitoring, as shown in Figure 5. For each case, three measurements were conducted to improve the reliability of the test.

Table 2 summarizes the data loss of each measurement. It can be seen that the data loss of uneven surface is higher than that of flat surface. After mounting the metal mesh, the data loss rate increased significantly. Therefore, the surface flatness has an important influence on the use of full cross-sectional laser scanner, and a high-level flatness is favorable in the application of the developed full cross-sectional deformation monitoring system.

**Figure 6:**
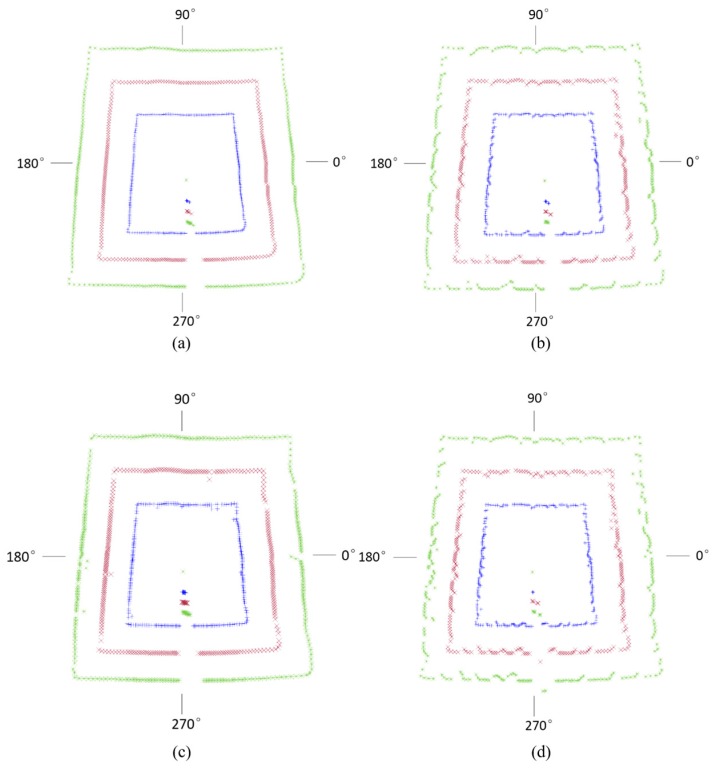
Measured data point distribution along the gateroad profile: (**a**) flat surface; (**b**) uneven surface; (**c**) flat surface with metal mesh; (**d**) uneven surface with metal mesh.

Figure 6 shows the measured data point distribution along the gateroad profile. For each level of flateness, the results of three measurements were plotted at a ratio of 1:1.5:2 for comparative purposes. It shows that the data loss of flat gateroad surface mainly occurred at the location of 277°–283°, uneven gateroads surface 277°–286°, flat gateroads surface with metal mesh 39°, 128°, 220°–224°, 281°, 327°, 333°, and uneven surface with metal mesh 139°, 275°–282°. It can be seen that the uneven flatness of gateroad profile can increase the incidence angle, finally resulting in an increase in the reflection angle of the laser beam. As a consequence, the reflected laser beam failed to reach the receiver. The abnormal result of 280° is due to the tripod shelter, as shown in Figure 6, and hence cannot be avoided.

### 4.2. The Influence of Surface Roughnesss on Data Loss

**Figure 7:**
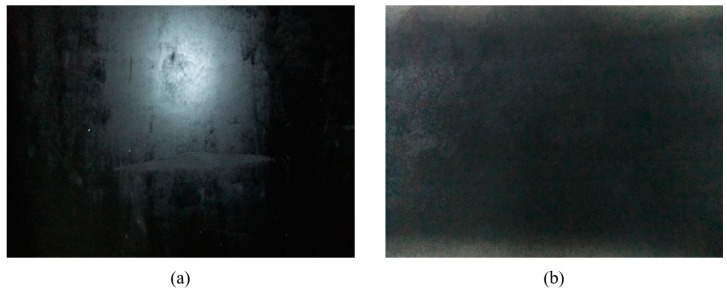
The surface roughness experiment of gateroads with developed deformation monitoring system: (**a**) smooth surface; (**b**) rough surface.

The surface roughness denotes the unevenness of the gateroad surface. Here, gateroads of smooth and rough surfaces were prepared to evaluate its influence on measurement data loss. The smooth and rough surfaces used are shown in Figure 7, while the gateroad geometric profile is exactly the same as Figure 3.

The experimental results of gateroad surface roughness is summarized in Table 3. It can be seen that the data loss of smooth surface is much more severe than that of rough surface case: the average percentage data loss of smooth surface is 38.93%, compared to 2.47% of the rough surface. The reason is that when meeting a relatively smooth target surface, the laser distance measurement sensor could not receive the reflected light when the incident angle of laser beam is large. However, when meeting a rough target surface, diffuse reflection occurs for laser beam, and laser distance measurement sensors can receive the diffused reflection light.

**Figure 8:**
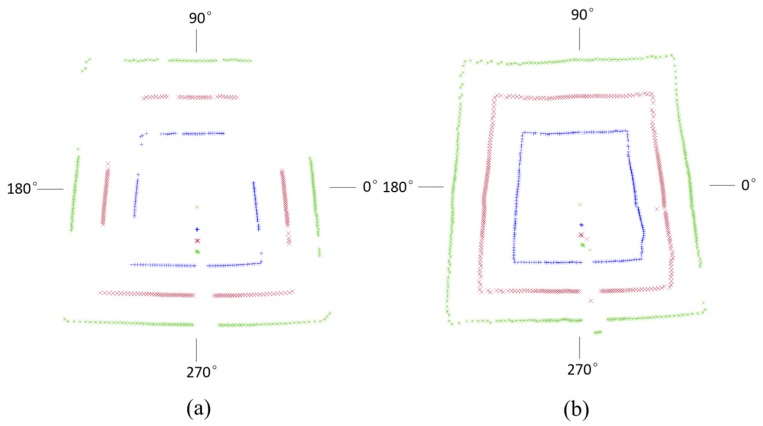
Measured data point distribution along the gateroad profile: (**a**) smooth surface; (**b**) rough surface.

Figure 8 shows the data loss of gateroads of smooth and rough surfaces. It indicates that the data loss of gateroad of smooth surface occurred at the 26°–71°, 119°–158°, 194°–222°, 275°–282°, and 324°–339°. The data loss of rough gateroad surface focused on 276°–285°, and 324°–334°. This implies that when the incident angle of laser beam is more than 70°, the developed full cross-sectional laser scanner is almost impossible to measure the distance information of smooth gateroad surface and the data loss rate is high. However, when gateroad surface is rough, the incident angle of laser beam has little effect on the use of the developed full cross-sectional laser scanner. Considering that the gateroads developed in coal mining are usually of a rough surface due to their short-term service time, the developed laser scanner can thus be well applied for the deformation measurement of underground gateroads.

### 4.3. The Influence of Gateroad Profile on Data Loss

In order to examine the performance of the developed laser scanner at gateroads of different geometric profiles, gateroads of trapezoidal, semi-circular arch and circular profile shapes were prepared, and measurements were conducted. The geometric profiles of three gateroads can be seen in Figure 3.

Table 4 shows the experimental results at gateroads of different surface roughness. It can be seen that gateroad geometric profile has a certain degree of influence on the application of full cross-sectional laser scanner: the data loss rate of trapezoidal opening profile is largest, and the data loss of circular gateroads is the least.

**Figure 9:**
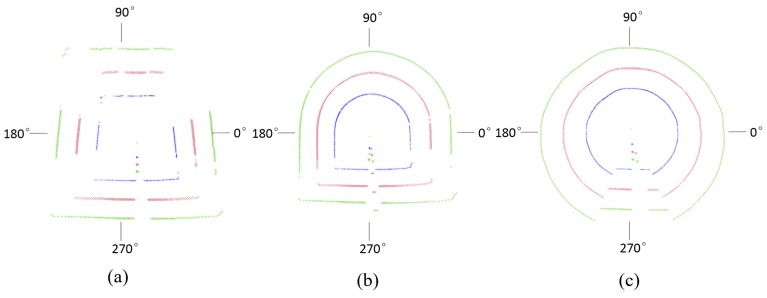
Measured data point distribution along the gateroad profile: (**a**) trapezoidal gateroads: (**b**) semi-circular arch gateroads; (**c**) circular gateroads.

The data loss distribution for gateroads of different geometric profiles is visually depicted in Figure 9. The data loss of trapezoidal gateroad mainly occurred at the locations of 26°–71°, 119°–158°, 194°–222°, 275°–282°, 324°–339°, semi-circular arch gateroad at 213°–224° and 330°–349°, and circular gateroad at 275°–279°. Therefore, measurement data in gateroad corners tends be lost. Such data loss at gateroad corners is also due to the increase of incidence angle, as at gateroad corners of small boundary curvatures the reflected laser beam could not be received by the laser scanner. Therefore, a high boundary curvature of gateroad geometric profile is favorable in using the full cross-sectional deformation monitoring laser scanner.

## 5. Influence of Environmental Coal Dust on Data Loss

**Figure 10:**
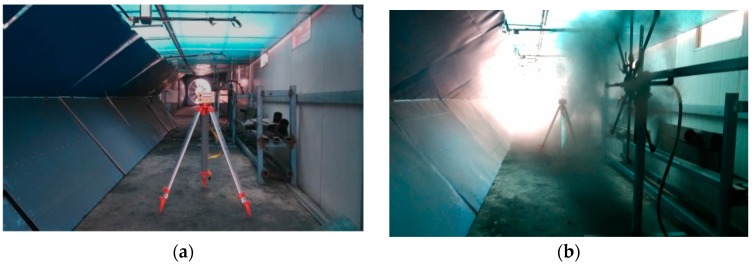
The experiment at gateroads of different dust concentration: (**a**) no coal dust; (**b**) a high level of coal dust.

In gateroads near a coal mining face or with a coal belt conveyor, coal dust cannot be avoided due to production operation [54,55,56]. The floating coal dust may also influence the deformation monitoring with this laser scanner. In order to evaluate its potential influence, gateroads with and without coal dust were reproduced, and measurements were conducted subsequently, as shown in Figure 10. During the experiment with coal dust, the lens of the laser displacement sensor can be blocked by dust fall and the experiment was performed after lens clean up.

Table 5 presents the experimental results at gateroads with and without coal dust. It can be seen that a large amount of data was lost when measurement was conducted under a high dust concentration: the average percentage data loss of high dust environment is 36.46%, compared to 15.23% for no coal dust case.

**Figure 11:**
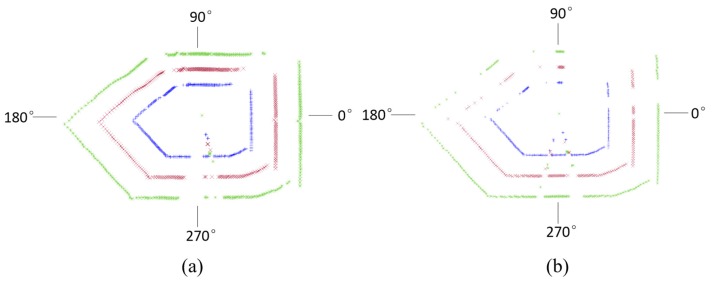
Measured data point distribution at gateroads of different degree of coal dust: (**a**) no coal dust; (**b**) a high level of coal dust.

Figure 11 shows measured data point distribution at gateroads with and without coal dust. It shows that significant data loss occurred under the condition of high dust concentration. Another phenomenon is that there is more data loss on the left-hand side of gateroads. However, this is mainly due to the fact that the dust distribution is uneven and tends to be random in the process of an experiment. The data loss mechanism is that the fine particles of floating coal dust leads to the scattering of the laser beam. As a result, the received reflection laser beam was significantly reduced and caused the loss of measurement data.

## 6. Conclusions

Based, considering the factors of gateroad wall flatness, gateroad wall roughness, gateroad geometric profile, and coal dust. It shows that a high-level flatness and roughness is favorable in application on the polar coordinate measuring method, a full cross-sectional deformation monitoring system was developed, which includes a visualization software package that can generate deformation profile of gateroads at different time instants. In order to evaluate its performance in practical application, the influence of complex environments of underground gateroads on data loss of laser distance measurement sensors was experimentally investigatedof the developed full cross-sectional deformation monitoring system. Further, high boundary curvature of gateroads can promote the reflection rate of emitted laser beam to the laser sensor, which is good for the practical measurement. However, fine particles of floating coal dust in the air can cause the loss of measurement data due to the scattering of the laser beam.

## Figures and Tables

**Table 1:** Laser displacement sensor parameters.

Electrical Parameter	Optical Parameter
Range resolution: 1 mm	Laser characteristics: Red laser diode
Measuring accuracy: 1.5 mm	Wavelength: 620 nm–690 nm
Maximum range: 10 m	Spot type: Single point
Operating temperature: −10 °C–50 °C	Single point accuracy: 6 mm at 10 m
Storage temperature: −30 °C–70 °C	Laser class: Laser class 2

**Table 2:** Data loss of each measurement at different levels of surface flatness.

Level of Flatness	The Number of Data Loss			Total Number of Lost Data	Percentage of Data Loss (%)
	First Measurement	Second Measurement	Third Measurement		
Flat surface	2	3	1	6	0.78
Uneven surface	2	3	5	10	1.30
Flat surface with metal mesh	7	8	3	18	2.34
Uneven surface with metal mesh	7	6	4	21	2.73

**Table 3:** The experimental results of gateroads surface roughens.

Level of Roughness	The Number of Data Loss			Total Number of Lost Data	Percentage of Data Loss (%)
	First Measurement	Second Measurement	Third Measurement		
Smooth surface	97	105	97	299	38.93
Rough surface	7	4	8	19	2.47

**Table 4:** The experimental results at gateroads of different geometric profiles.

Geometric Profile	The Number of Data Loss			Total Number of Lost Data	Percentage of Data Loss (%)
	First Measurement	Second Measurement	Third Measurement		
Trapezoidal gateroad	97	105	97	299	38.93
Semi-circular arch gateroads	20	20	25	70	9.11
Circular gateroads	5	5	4	13	1.69

**Table 5:** The experimental results at gateroads with and without coal dust.

Environment	The Number of Data Loss			Total Number of Lost Data	Percentage of Data Loss (%)
	First Measurement	Second Measurement	Third Measurement		
No coal dust	38	39	40	117	15.23
A high level of coal dust	90	96	94	280	36.46
